# Mortality differences between migrants and Italians residing in Rome before, during, and in the aftermath of the great recession. A longitudinal cohort study from 2001 to 2015

**DOI:** 10.1186/s12889-021-12176-8

**Published:** 2021-11-17

**Authors:** Eleonora Trappolini, Claudia Marino, Nera Agabiti, Cristina Giudici, Marina Davoli, Laura Cacciani

**Affiliations:** 1 University of Milano-Bicocca, Milan, Italy; 2 Department of Epidemiology, Lazio Regional Health Service, Via Cristoforo Colombo, 112, 00147 Rome, Italy; 3 Sapienza University of Rome, Rome, Italy

**Keywords:** Mortality, Migrants, Longitudinal study, Dynamic cohort, Great recession, Italy

## Abstract

**Background:**

In Europe, one of the most consistent findings is that of migrant mortality advantage in high-income countries. Furthermore, the literature shows that economic shocks, which bring worse health outcomes, can severely affect the most disadvantaged individuals. We analyse differences and changes in all-cause mortality between Italians and migrants residing in Rome before, during, and in the aftermath of the Great Recession (2001–2015) by birth-cohort.

**Methods:**

The analysis is a longitudinal open cohort study. Mortality data come from the Register of the Causes of Death (58,637 deaths) and the population denominator (*n* = 2,454,410) comes from the Municipal Register of Rome. By comparing three time-periods (2001–2005, 2006–2010, and 2011–2015), we analyse all-cause mortality of Rome residents born, respectively, in the intervals 1937–1976, 1942–1981, 1947–1986 (aged 25–64 years at entry into observation). Computing birth-cohort-specific death rates and applying parametric survival models with age as the time-scale, we compare mortality differences between migrants and Italians by gender, area of origin, and time-period.

**Results:**

Overall, we find a lower risk of dying for migrants than Italians regardless of gender (Women: HR = 0.61, 95% CI 0.56–0.66; Men: HR = 0.49, 95% CI 0.45–0.53), and a lower death risk over time for the total population. Nevertheless, such a pattern changes according to gender and migrants’ area of origin.

**Conclusion:**

Given the relevance of international migrations in Europe, studying migrants’ health has proved increasingly important. The deterioration in migrant health and the gradual weakening of migrants’ mortality advantage is likely to become a public health issue with important consequences for the healthcare system of all European countries.

**Supplementary Information:**

The online version contains supplementary material available at 10.1186/s12889-021-12176-8.

## Background

In the past 50 years, health improvements have been registered all over Europe. However, there are still many examples of mortality differences by social group [1, 2], by gender [3, 4], and for migrants compared with host populations [5–7].

The 2008 Great Recession, which was the result of the financial crisis that started in late 2007 in the United States, was particularly severe in Southern Europe, where there were big increases in unemployment, as well as generalized banking problems, large public and private debts, and austerity policies. In Italy, the negative growth registered in the GDP has been stronger compared to other Eurozone countries, and when the second phase of the crisis started, known as the European sovereign debt crisis, the Country again entered into recession starting from July 2011 [8], continuing to experience negative economic growth until the whole 2014 [9].

Among other things, the recession caused serious financial issues for public services, including the healthcare sector. Reductions in healthcare spending compromised the quality of services provided, leading to a deterioration in health outcomes [10]. Moreover, reductions in household budgets due to unemployment, or reductions in pensions and wages, decrease the individual’s ability to adopt healthy lifestyles and pay for healthcare, again leading to a deterioration in health. This deterioration seems to be particularly evident in countries with weak social protection [11].

In Europe, the impact of the Great Recession on health remains controversial and empirical studies on health and mortality differ in their findings [12–18]. Counter-intuitively, at the population-level, many authors have reported a pro-cyclical effect on general mortality, known as the “Thomas effect”, which means mortality goes up with economic expansions and down with contractions [19–28]. Conversely, at the individual-level, some scholars have registered quite the contrary. Whatever the health indicator used, the effect of economic downturns is always associated with a deterioration in health outcomes [29, 30]. In Italy, Egidi and Demuru (2016) [31] claimed that the Great Recession caused a slowdown in mortality improvements.

Regarding differences in mortality between natives and migrants, one consistent finding in the literature relates to the migrant mortality advantage in high-income countries. This phenomenon implies that migrants have lower mortality than natives or at least lower than might be expected given their double disadvantage, as migrants and their relative poverty [5–7, 32–36].

Although in Italy the number of migrants has constantly increased, reaching a peak of more than 5.2 million in 2019 (8.7% of the total population) [37], there are few studies on migrant mortality. There are a handful of cross-sectional works [38–40] and only two longitudinal studies [41, 42]. As far as we know there are no studies on mortality differences between migrants and non-migrants by birth-cohort before and during the Great Recession.

Our study contributes to the Italian literature about migrants’ mortality which is still poor. It does so by providing additional evidence on mortality differences between migrants and Italians during the economic crisis. Using a longitudinal approach based on a dynamic population cohort, the aim of this study is twofold. First, it analyses all-cause mortality differences between Italians and migrants residing in Rome before and during the Great Recession (2001–2015) by birth-cohort. Second, by comparing three different five-year time-periods (2001–2005, 2006–2010, and 2011–2015) the study explores whether, comparing mortality among migrants with mortality among the Italian-born population, hazard ratios differ in the time-periods analysed.

Given the mortality advantage observed in high-income European countries among migrants, our first hypothesis is that since, in Italy, the migration phenomenon is relatively recent compared to other European countries, the healthy migrant effect will still be evident. Migrants may benefit, then, from a survival advantage. The analysis over a period of 15 years allows to assess changes in mortality patterns by migrant status. We expect to find a decrease in mortality level for Italians, following the pattern of improvement observed in the last years. We also expect to find a decrease in the mortality advantage for migrants because both of their low socio-economic condition, which persists through the whole period under analysis; and the increase in migrants’ length of stay, associated with negative acculturation and assimilation processes, compounded by the economic downturn. Finally, we hypothesise that the effect of the economic downturn on mortality will be mostly captured during the third period, which combined the effects of the first and second phases of the crisis.

## Methods

### The study setting

The study was set in Rome, the capital of Italy, which is situated in the Lazio region in central Italy. In 2019, the city counts 2.8 million residents, and it is the first Italian city by number of migrants (more than 340,000). In the last two decades, the city was characterised by a rapid increase in the migrant population, which passed from 3.9% of the total residents in 2002 to 13.4% in 2019 [37]. Currently, the largest migrant communities settled in Rome are Romanians and Filipinos, which are female-dominated. Indeed, in Rome female migrants represented 53% of the total migrants. This aspect may show the tendency of the capital to attract female migrants, particularly for domestic jobs [43]. As at the national level, another characteristic of the city is to host a plurality of migrants’ country of origin, about 195 different nationalities in 2019 [37], which reflect different cultures, behaviours, and health needs.

### Research design and cohort description

A longitudinal population-based open cohort study was conducted, using data from the Rome Dynamic Longitudinal Study cohort, which is part of the Italian Network of Longitudinal Metropolitan studies (IN-liMeS) [41, 42, 44]. The dynamic cohort is based on the Municipal Register of Rome, which provides individual demographic information (sex, age, birthdate, birthplace, date of registration in the Municipality of Rome, and date of cancellation from the population register) for all who have been resident in Rome from 1st January 2001 to 31st December 2015 (4,143,462 records, which correspond to 3,978,400 individuals).^1^

Starting from the cohort data, we excluded records that did not have reliable Health Information System code (43,393 out of 4,143,462, 1.0%), for a total of 3,935,007 individuals. For analysis purposes, we identified three time-periods (2001–2005, 2006–2010, 2011–2015) allowing for a maximum follow-up of five years in each time-period, and for a maximum of 15 years overall. At the beginning of each time-period, we considered residents in Rome aged 25–64 years. Thus, for the first period we selected those individuals belonging to the birth cohorts from 1937 to 1976 (1,822,603 individuals); for the second time-period those from 1942 to 1981 (1,842,393 individuals); and for the third one those from 1947 to 1986 (1,815,152 individuals). Within each time-period, entry into the study population can take place for immigration or age (≥ 25 years old). Meanwhile, exit can come about because of emigration, age (≥ 65 years old), death, or the end of the study. Subsequently, we computed person-years at risk from the date of enrolment until death, emigration, or to the end of the follow-up. Combining the three time-periods the final study population is composed of 2,454,410 individuals.

### Study variables

#### Outcome

The outcome variable is all-cause mortality, retrieved by linking the cohort data with the register of the causes of death (ReNCaM) using an individual anonymised code. The register contains information about the deaths of all residents in the Lazio region, in which Rome is to be found.

#### Exposures and control variables

Migrant status is the exposure variable (migrants vs. Italians). This study defined as migrant all individuals born abroad. For more in-depth analyses we classified migrants according to their area of origin, distinguishing between migrants coming from High Migratory Pressure Countries (HMPC: Central Eastern Europe, Africa, Asia – except for Israel and Japan – and Latin America) and migrants coming from all other countries, i.e. Highly Developed Countries (HDC). Being Italian is the reference category.

The time-period is considered as a potential effect modifier. It takes the value ‘0’ from 01/01/2001 to 31/12/2005, ‘1’ from 01/01/2006 to 31/12/2010, and ‘2’ from 01/01/2011 to 31/12/2015.

Gender is a stratification variable.

### Statistical analysis

In order to analyse differences and changes in mortality between migrants and non-migrants, in the first part of the study, we investigated mortality patterns by time-period (2001–2005, 2006–2010, 2011–2015), by considering different birth-cohorts of residents in Rome by gender. Combining death and person-years, we computed birth-cohort-specific death rates by gender, time-period, and migrant status.

In the second part of the study, we used a parametric survival model with Gompertz baseline hazard to examine the influence of the area of origin on mortality. In the case of mortality, a Gompertz distribution (which suggests an exponential increase in mortality over age) has been shown to provide a very close fit to adult mortality in western countries [5]. We used age as time-scale as it modifies an individual’s risk of dying [45]. For our purposes, death is the failure event. So individuals enter the analysis at their baseline age (left-truncation) and exit at their failure event or censoring age, emigration, or the end of the follow-up.

To test whether the time-period effect on all-cause mortality differs among migrant subgroups we included the interaction between the area of origin and the time-period.

The SAS software environment 9.4 was used for data management, and all calculations have been performed using STATA 15.

## Results

We included 2,454,410 individuals who resided in Rome from 2001 to 2015; among them, 19.7% were migrants (*n* = 484,421), of whom 53.3% women. Over the study period, we observed 58,637 deaths within 24.6 million person-years; among migrants, 3766 deaths (6.4% of all deaths) occurred within 3.6 million person-years. The migrant population came mainly from Central Eastern Europe, with an increase from 2.7% (2001–2005) to 7.1% (2011–2015), followed by, Asia, Africa, HDC, and Latin America. As in the national context, migrants mitigate the ageing of the Roman population: over the study period the median age among migrants was 39.4 years old vs. 43.1 among Italians. Table 1 shows descriptive statistics of the socio-demographic characteristics included in the analysis of migrant groups and the Italian-born population by the three time-periods separately.

**Table 1:** Demographic characteristics of migrants and Italians residing in Rome, and deaths by time-period

	2001–2005				2006–2010				2011–2015			
	Italians		Migrants		Italians		Migrants		Italians		Migrants	
	Subjects^**a**^	Deaths^**b**^	Subjects	Deaths	Subjects	Deaths	Subjects	Deaths	Subjects	Deaths	Subjects	Deaths
**Area of origin**												
Italy	86.8	95.0	–^c^	–	82.1	93.8	–	–	78.6	91.5	–	–
HDC	–	–	2.8	1.1	–	–	2.9	1.1	–	–	2.6	1.1
HMPC	–	–	10.4	3.9	–	–	15.0	5.1	–	–	18.8	7.4
*of which*												
*Africa*	–	–	* 2.5*	* 1.9*	–	–	* 2.7*	* 1.8*	–	–	* 3.2*	* 2.0*
*Latin America*	–	–	* 2.2*	* 0.6*	–	–	* 2.7*	* 0.7*	–	–	* 2.6*	* 1.0*
*Asia*	–	–	* 3.0*	* 0.7*	–	–	* 4.1*	* 1.2*	–	–	* 5.9*	* 1.8*
*Central Eastern Europe*	–	–	* 2.7*	* 0.7*	–	–	* 5.5*	* 1.4*	–	–	* 7.1*	* 2.6*
**Gender**												
Women	44.0	35.4	7.2	2.3	41.6	36.3	9.8	2.7	40.0	36.3	11.4	3.8
Men	42.7	59.6	6.0	2.6	40.5	57.6	8.1	3.5	38.7	55.2	10.0	4.7
**Birth Cohort**												
1982–1986	–	–	–	–	–	–	–	–	7.3	1.1	3.2	0.3
1977–1981	–	–	–	–	8.3	1.8	2.9	0.2	8.6	1.8	3.7	0.5
1972–1976	11.3	2.1	2.2	0.2	11.3	2.6	3.2	0.4	11.0	3.3	3.5	0.7
1967–1971	12.6	3.4	2.6	0.4	12.2	4.4	3.2	0.6	11.7	6.0	3.3	0.9
1962–1966	12.9	5.0	2.4	0.4	12.4	7.1	2.7	0.8	12.0	10.6	2.7	1.1
1957–1961	10.9	6.7	1.9	0.5	10.4	9.7	2.2	0.8	10.1	14.8	2.2	1.5
1952–1956	9.7	9.3	1.5	0.6	9.3	14.1	1.7	1.0	9.0	21.1	1.6	1.8
1947–1951	10.0	13.9	1.1	0.7	9.4	21.8	1.2	1.2	8.9	32.8	1.1	1.8
1942–1946	9.4	20.8	0.8	0.8	8.8	32.3	0.8	1.1	–	–	–	–
1937–1941	9.9	34.0	0.7	1.3	–	–	–	–	–	–	–	–
	**2001–2005**				**2006–2010**				**2011–2015**			
**Total population**^**d**^	** 1,822,603**				** 1,842,393**				** 1,815,152**			
	** Italian**		** Foreign**		** Italian**		** Foreign**		** Italian**	** Foreign**		
	* 1,581,282*		* 241,321*		*1,512,537*		*329,856*		* 1,427,233*	* 387,919*		
**Total deaths**^**d**^	** 22,214**				** 18,440**				** 17,983**			
	** Italian**		** Foreign**		** Italian**		** Foreign**		** Italian**	** Foreign**		
	* 21,113*		* 1101*		* 17,304*		* 1136*		* 16,454*	* 1529*		

*Notes*: HDC: Highly Developed Countries; HMPC: High Migratory Pressure Countries

^a^Subjects in percentages, out of the total population: e.g., out of the total population (1,822,603 in 2001–2005) 86.8% are Italians

^b^Deaths in percentages, out of the total deaths: e.g., out of the total deaths (22,214 in 2001–2005) 95.0% occurred among Italians

^c^Not applicable

^d^In absolute numbers

*Source:* Authors’ elaboration on Dynamic Rome Longitudinal Study cohort data and the Register of causes of death (ReNCaM)

**Fig. 1:**
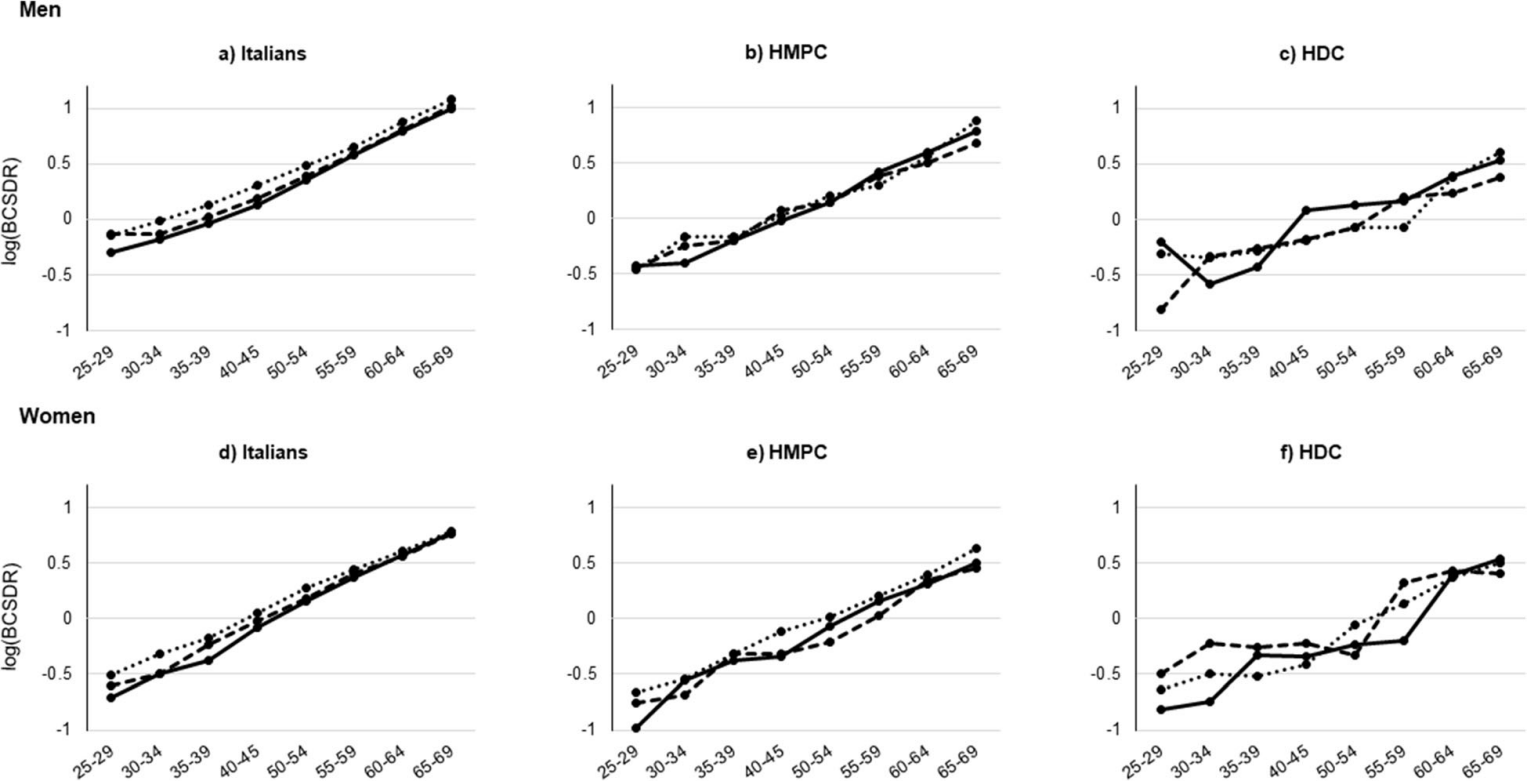
Birth-cohort-specific death rates (BCSDR) by gender, migrant status, and time-period, 2001–2015. *Notes:* For the 2001–2005 time-period we selected the birth-cohorts from 1937 to 1976; for the 2006–2010 time-period the birth-cohorts from 1942 to 1981; for the 2011–2015 time-period the birth-cohorts from 1947 to 1986. In Appendix A, Fig. 1A shows birth-cohort-specific death rates (BCSDR) on a log-scale by gender, time-period, and migrant status. *Source:* Authors’ elaboration on Dynamic Rome Longitudinal Study cohort data and the Register of causes of death (ReNCaM)

Figure 1 shows birth-cohort-specific death rates (BCSDR) on a log-scale by gender, migrant status, and time-period. Migrants showed lower BCSDR compared with Italians in each time-period. When comparing individuals of the same age, a decrease in the BCSDR can be detected among Italians, particularly among men and at young and adult ages, while such pattern is not clearly detected for migrants. Overall, women display lower BCSDR than men in all three time-periods analysed.

### Regression results for migrants as a heterogeneous group versus natives

Table 2 shows the hazard ratios (HR) in gender-specific populations controlling for migrants from different areas of origin as a single heterogeneous group, time-period, and an interaction term between migrant status and the time-period in order to test whether the time-period effect on all-cause mortality differs between Italian and migrants.^2^ We found that the adjusted risk of dying for female (HR = 0.61, 95% CI 0.56–0.66) and male (HR = 0.49, 95% CI 0.45–0.53) migrants is lower compared to the risk for natives. Compared to the first time-period (2001–2005), results show lower mortality in the second (2006–2010: Women, HR = 0.89, 95% CI 0.86–0.92 -- Men, HR = 0.84, 95% CI 0.81–0.86) and third time-period (2011–2015: Women, HR = 0.86, 95% CI 0.83–0.89 -- Men, HR = 0.77, 95% CI 0.75–0.79) for both genders. In addition, from a further analysis that we do not show for sake of brevity, switching and choosing the 2006–2010 time-period as the reference category, we found that the difference in mortality between the third and the second time-period is smaller than that between the second and the first time-period. Specifically, among men the difference (between the third and the second time-period) is small (HR = 0.92, 95% CI 0.90–0.95, *p*-value < 0.001), while among women, although the difference is still detectable, it is slightly significant (HR = 0.97, 95% CI 0.94–1.00, *p*-value 0.067). This result might suggest a slowdown in mortality decrease.

**Table 2:** Gender-specific mortality HR for migrants versus Italians residing in Rome, 2001–2015

	Women			Men		
	HR		95% CI	HR		95% CI
**Migrant status**						
Italian	1.00			1.00		
Migrant	0.61	***	(0.555–0.663)	0.49	***	(0.452–0.534)
**Time-period**						
2001–2005	1.00			1.00		
2006–2010	0.89	***	(0.860–0.918)	0.84	***	(0.814–0.857)
2011–2015	0.86	***	(0.834–0.890)	0.77	***	(0.750–0.790)
**Interaction:** Migrant status*Time-period^**a**^						
Migrant 2006–2010	0.84	**	(0.743–0.958)	1.07		(0.951–1.197)
Migrant 2011–2015	0.95		(0.843–1.069)	1.27	***	(1.141–1.419)
*N observations*	* 2,843,960*			* 2,704,604*		

*Notes:* Parametric survival model with Gompertz baseline hazard and age as the time-scale

The asterisks indicate significance *p < 0.05, **p < 0.01, ***p < 0.001

^a^Likelihood ratio test – Women: *p*-value = 0.026; Men: *p*-value = 0.000

*Source:* Authors’ elaboration on Dynamic Rome Longitudinal Study cohort data and the Register of causes of death (ReNCaM)

As regards the interaction between migrant status and the time-period, the results suggest that in 2011–2015 the effect of time on mortality is less strong on migrant men than the average effect of time on Italians, while there are no differences in 2006–2010. Conversely, among migrant women, the effect of time on mortality is stronger in 2006–2010, without differences in the third time-period.

### Regression results by migrants’ area of origin

**Fig. 2:**
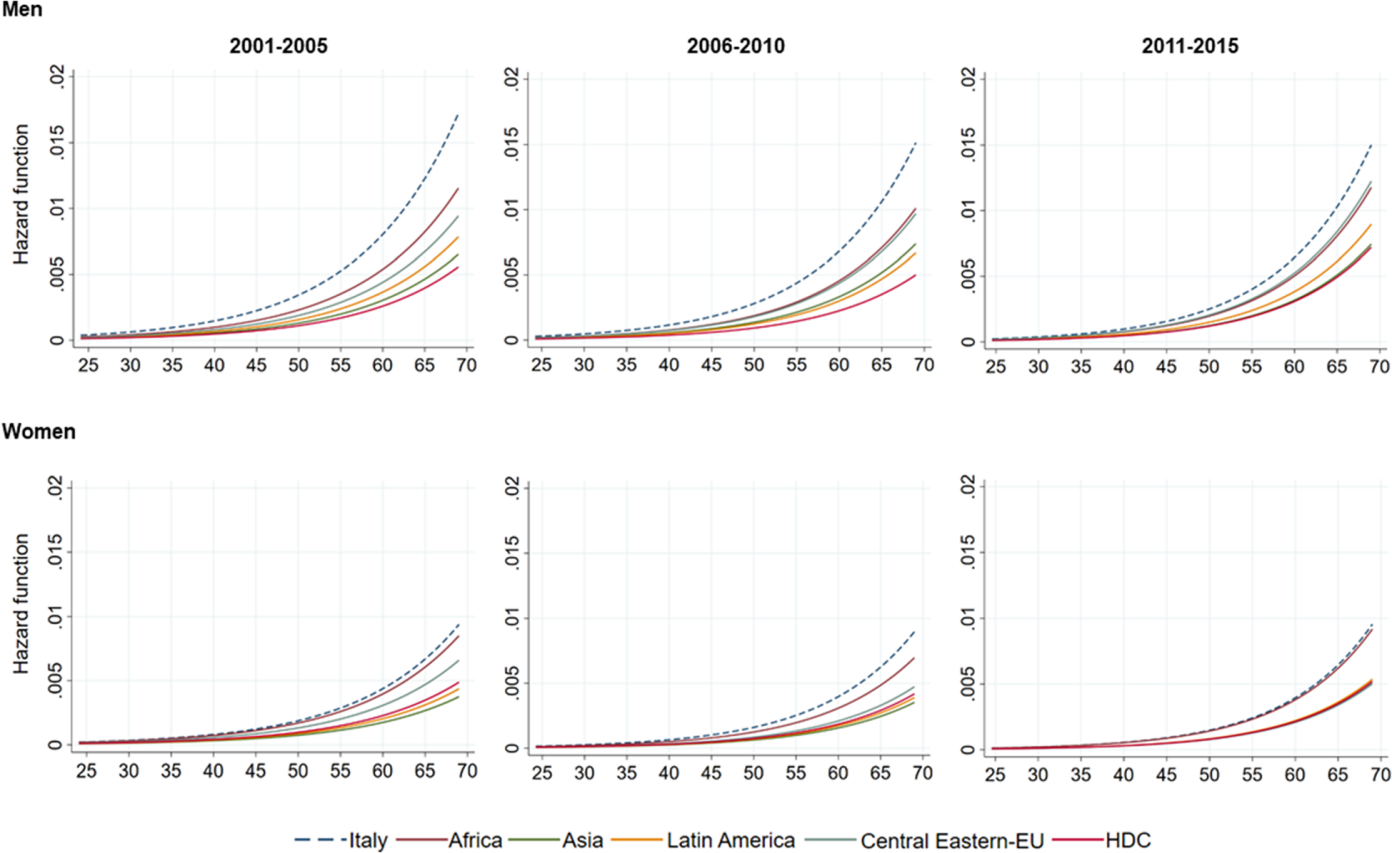
Hazard estimates by gender, time-period, and area of origin. Residents in Rome, 2001–2015. *Source:* Authors’ elaboration on Dynamic Rome Longitudinal Study cohort data and the Register of causes of death (ReNCaM)

Figure 2 shows gender-specific hazard estimates by time-period and by area of origin. All migrant groups have lower mortality with respect to Italians (blue dot line) for both men and women, across age and across time-period. The graph also displays changes over time in the risk of dying for the populations considered. Looking at the three time-periods separately, for male and female Italians, hazard curves suggest a lower risk of dying over time. Actually, from the first time-period to the third one, the blue dot line falls, suggesting an increase in survival probability. Conversely, the coloured solid lines, which represent migrant groups, get closer with time, showing a reduction in the mortality gap between Italians and migrants, in particular among migrant men. In addition, for both men and women (except for African women) the mortality difference between Italians and migrant groups was always statistically significant (Table 3). Among women, the mortality pattern in the 2011–2015 time-period shows two clusters: Italians and Africans had a similar risk of dying (the hazard curves overlap); while Asians, Central Eastern Europeans, Latin America, and HDC had a mortality advantage.

**Table 3:** Gender-specific mortality HR for different migrant groups versus Italians residing in Rome, 2001–2015

	Women			Men		
Area of origin	HR		95% CI	HR		95% CI
Italy	1.00			1.00		
Africa	0.91		(0.780–1.058)	0.67	***	(0.594–0.764)
Asia	0.41	***	(0.321–0.530)	0.39	***	(0.318–0.472)
Latin America	0.48	***	(0.373–0.611)	0.46	***	(0.364–0.592)
Central-Eastern Europe	0.73	**	(0.582–0.903)	0.56	***	(0.452–0.703)
HDC	0.52	***	(0.442–0.615)	0.33	***	(0.265–0.400)
**Time-period**						
2001–2005	1.00			1.00		
2006–2010	0.89	***	(0.860–0.918)	0.84	***	(0.814–0.857)
2011–2015	0.86	***	(0.834–0.890)	0.77	***	(0.750–0.790)
**Interaction:** Area of origin*Time-period^**a**^						
Africa 2006	0.85		(0.675–1.083)	0.99		(0.822–1.193)
Africa 2011	1.05		(0.839–1.322)	1.15		(0.958–1.380)
Asia 2006	0.95		(0.679–1.335)	1.26		(0.977–1.622)
Asia 2011	1.24		(0.913–1.681)	1.24		(0.971–1.582)
Latin America 2006	0.91		(0.642–1.291)	0.95		(0.666–1.359)
Latin America 2011	1.15		(0.838–1.576)	1.26		(0.910–1.745)
Central Eastern Europe 2006	0.73	*	(0.548–0.963)	1.14		(0.862–1.498)
Central Eastern Europe 2011	0.71	**	(0.546–0.913)	1.39	**	(1.080–1.797)
HDC 2006	0.89		(0.696–1.146)	1.01		(0.753–1.368)
HDC 2011	1.04		(0.804–1.334)	1.46	**	(1.096–1.944)
*N observations*	* 2,843,960*			* 2,704,604*		

*Notes:* Parametric survival model with Gompertz baseline hazard and age as the time-scale

The asterisks indicate significance *p < 0.05, **p < 0.01, ***p < 0.001

^a^Likelihood ratio test – Women: *p*-value = 0.044; Men: *p*-value = 0.004

In Appendix B, Table 2B shows gender-specific mortality HR for migrants’ area of origin versus Italians residing in Rome stratified by time-period

*Source:* Authors’ elaboration on Dynamic Rome Longitudinal Study cohort data and the Register of causes of death (ReNCaM)

Combining data for the different time-periods we examined gender-specific hazard ratios for each migrant group according to the area of origin. Mortality among women from Asia, Latin America, Central Eastern Europe and HDC was, respectively, 59% (HR = 0.41, 95% CI 0.32–0.53), 52% (HR = 0.48, 95% CI 0.37–0.61), 27% (HR = 0.73, 95% CI 0.58–0.90) and 48% (HR = 0.52, 95% CI 0.44–0.62) lower than Italians; while mortality among women from Africa was similar to mortality among Italian women (HR = 0.91, 95% CI 0.78–1.06). Mortality among men from Africa, Asia, Latin America, Central Eastern Europe and HDC was, respectively, 33% (HR = 0.67, 95% CI 0.59–0.76), 61% (HR = 0.39, 95% CI 0.32–0.47), 54% (HR = 0.46, 95% CI 0.36–0.59), 44% (HR = 0.56, 95% CI 0.45–0.70) and 67% (HR = 0.33, 95% CI 0.27–0.40) lower than their Italians counterparts. Compared to 2001–2005, in the second (2006–2010) and third (2011–2015) time-period, a lower risk of dying was observed for the whole population, regardless of gender, with some differences according to the area of origin. Among men, the interaction between the area of origin and time-period suggests that the effect of time on their death risk was less strong among Central Eastern Europeans in the 2011–2015 period and among migrants from HDC in the 2011–2015 period. Conversely, among women, the effect of time on mortality was stronger only among Central Eastern Europeans in the 2006–2010 and in the 2011–2015 period, while there were no changes in the risk of dying over time, between Italians and all other migrant groups (Table 3).^3^

## Discussion

Using a population-based open cohort design, the present study investigates the association between migrant status and all-cause mortality among Rome residents from 2001 to 2015 by birth-cohort. By comparing three different time-periods, we also analyse changes in mortality patterns before and during the Great Recession.

In line with other international [5, 6, 46–53] and national [38–42] studies, the findings confirm the first hypothesis on the migrant mortality advantage. Compared to the Italian-born population, migrants have significantly lower all-cause mortality, regardless of the area of origin, and for both genders; except for African women who register similar mortality patterns to Italian women. The migrant mortality advantage may result from two selection hypotheses. The first one, known as the healthy migrant effect [46, 54, 55], suggests the selection of healthy individuals into migration. The second one, known as the salmon bias [56, 57], proposes the remigration of unhealthy individuals to their origin country, something particularly important among elderly migrants. However, the remigration of unhealthy individuals to their origin country is hard to test and few existing studies analyse this issue. Norredam et al. (2014) [58], for instance, found weak support for the remigration bias hypothesis in Denmark. Recently, in another study by Di Napoli et al. (2021) [59], which analyse the salmon bias effect hypothesis among migrants in Italy, the authors found that the salmon bias only partly explains the difference in mortality rates between migrants and non-migrants.

What is more, the data artefact and its reliability should be taken into account [5, 60–62]. We have delays in registration in municipal registries upon arrival or the final return to the origin country, which can lead to an additional underestimation of migrants’ mortality. In a period of economic crisis, the increased mobility of migrants who may leave the host country to look for job opportunities in other countries or remigrate to their origin country will perhaps affect the data artefact more strongly [63]. Nevertheless, Wallace and Wilson (2021) [64], studying the Swedish context, found that the data artefact could explain some, but not all, of the mortality advantage detected. This result demonstrates that such a pattern is real.

As expected, we found support for our second hypothesis about mortality changes over time. Overall, we found a lower risk of dying for both genders over time. This pattern reflects the general improvement detected in the last years, resulting in an increase in life expectancy [65, 66]. In addition, whereas during the time period analysed, the Italian economic conjuncture was particularly negative, one would have expected a mortality increase (counter-cyclical effect). The observed pro-cyclical effects (meaning mortality goes up with economic expansions and down with contractions), which is counterintuitive, should be referred to the “Thomas effect” [67]. The explanation for this pattern is that some important determinants of ill health and death are correlated with economic activity. For example, for trafficking-related mortality, economic downturns reduce industrial and commercial traffic, as well as commuting and recreational driving. Furthermore, we must also consider that economic expansion also brings overtime hours and higher intensity work, leading to less time for sleep, physical activity, and social interactions. Atmospheric pollution, social isolation, and cigarette smoking also increase in times of economic growth and decrease in recessions [68–70].

Finally, our findings support the third hypothesis according to which the effect of the Great Recession might be captured during the third period. This is in line with a recent study by Egidi and Demuru (2016) [31], where the authors studied the relationship between the economic crisis and mortality trends at the national level, revealing that the Great Recession had modified and slowed down mortality trends, in particular mortality improvements.

The effect of time detected for the whole population changes according to gender and area of origin. Among men, the mortality improvement was less strong for Central Eastern Europeans in the 2011–2015 time-period, and for migrants from HDC in the 2011–2015 time-period. This finding could be related to the often poor socioeconomic conditions in which migrants live [71, 72] and to the process of acculturation that may induce scarce opportunities to adopt healthy habits and lifestyles [73, 74]. It could also be explained by the economic downturn compounding precarious employment conditions and low socioeconomic status. Certainly, there is evidence that migrant health worsens with longer residence in the host country [44, 75–77]. However, in the absence of information about the length of stay, we cannot confirm this hypothesis. Conversely, among women, we found that mortality improvements were stronger only among Central Eastern Europeans both in the second (2006–2010) and the third (2011–2015) time-period with respect to the first (2001–2005). No difference was observed for other migrant groups. This trend could be related to EU membership for Poland and the Czech Republic in 2004 and for Romania and Bulgaria in 2007. This modified the composition of migration flows to Italy, helped along by strong Italian demand for domestic workers and caregivers [77, 78]. Finally, further studies are needed to investigate the socio-structural drivers of the differences in mortality that emerged in the analysis. As argued by Hossin (2020) [79], these differences are linked to a wide range of premigratory and postmigratory vulnerabilities that mutually intersect.

This study is not without limitations. We could not account for some important mortality risk factors. As previously mentioned, migrants' length of stay in the host country is an important confounder. Several studies conducted in France [80], Canada [81], Belgium [73], and Norway [74], have shown an increase in migrant mortality with an increase in the length of stay.

In addition, our study did not consider socio-economic variables, such as income, education, or even the reason for migration, which can influence the outcome and could have helped to explain the complex relationship between migrant status and mortality [82–85]. Furthermore, since the cohort is based on the Municipal Register of Rome, we included only residents, which means only regular migrants, while migrants without residence permits, who represent a particularly vulnerable population, were not included. Finally, the use of data from the Municipality of Rome might affect the external validity of the results because it is considered a part of the immigrant population residing in Italy. However, as regards the composition of migrantion flows, the distribution of the migrant population in Rome and in Italy is quite similar. In both cases, Romanians are the first community (36%), and about 50% of migrants come from Eastern Europe. The major differences concern migrants from the Philippines and Bangladesh who are over-represented in Rome and this might limit the external validity of the results [37].

If there are drawbacks this study also has, though, important strengths. First, by using a longitudinal approach with open cohort data we were able to enrol all new entries during the follow-up, reflecting the great dynamism of the migrant population. Second, by linking the Municipal Register of Rome to the Register of Causes of Death we computed person-years, allowing us to obtain the person-time at risk and to estimate accurately mortality rates. Finally, using age as the time-scale gives more accurate results because it puts similar subjects in the risk set together and allows a completely non-parametric age effect. Furthermore, each individual contributes to the death risk only in the age interval in which he/she is exposed to the risk of experiencing the event.

Our findings are relevant for contemporary health systems because, for the first time, Italy has to deal with a significant migrant population. Even if the study is context-specific, our analyses rely on the Municipality of Rome which has the highest number of migrants in Italy and, therefore, represents a relevant and useful context for studying this issue, and one useful for health policy makers thinking of migrant mortality trends. Moreover, by analysing a period over 15 years, from 2001 to 2015, this work helps to better understand mortality trends by birth-cohort and to provide insights into the mortality patterns during the Great Recession.

Given the relevance of international migrations all over Europe, the importance of studying and exploring migrants’ health is becoming increasingly evident. This is especially true for the implementation of targeted policies addressing migrants’ integration. The deterioration in migrant health and the gradual weakening of their mortality advantage is likely to become a public health issue with important consequences for the healthcare system of all European countries.

Further researches with a longer follow-up and analyses focused on causes of death would be needed for a better description and explanation of mortality differential patterns observed between migrants and non-migrants.

## Supplementary Information

**Additional file 1.**

## Data Availability

The datasets generated, used and/or analysed during the current study are not publicly available due to stringent legal restrictions regarding the privacy policy on personal information in Italy (national legislative decree on privacy policy n. 196/30 June 2003) and ethical reasons.

## Abbreviations

BCSDR: Birth-Cohort-Specific Death Rates
CI: Confidence Interval
HDC: Highly Developed Countries
HMPC: High Migratory Pressure Countries
HR: Hazard Ratios
IN-liMeS: Italian Network of Longitudinal Metropolitan studies
ReNCaM: Register of the Causes of Death

## Footnotes

1 Each row of the cohort dataset is a record that contains individual information from the population register of Rome. In the dynamic cohort, individuals may have multiple records as they can enter as residents in Rome, emigrate and remigrate afterwards.

2 In Appendix B, Table 1B shows gender-specific mortality HR for migrants versus Italians residing in Rome stratified by time-period. The likelihood ratio test shows that the model with the interaction term fits significantly better than the model without the interaction term for both genders (*p* < 0.05).

3 In Appendix B, Table 2B shows gender-specific mortality hazard ratios for different migrant groups versus Italians residing in Rome by time-period. The likelihood ratio test shows that the model with the interaction term fits significantly better than the model without the interaction term for both genders (*p* < 0.05).
