# Supplementary material for: Mortality differences between migrants and Italians residing in Rome before, during, and in the aftermath of the great recession. A longitudinal cohort study from 2001 to 2015
Source: BMC Public Health. 2021 Nov 17;21:2112. doi: 10.1186/s12889-021-12176-8 (PMC8600794; doi:10.1186/s12889-021-12176-8)
Supplement: Supplementary file 1 — Additional file 1. [file 12889_2021_12176_MOESM1_ESM.docx]

**Appendix A**

**Figure 1A. Birth-cohort specific death rates (BCSDR) by gender, time-period and migrant status.**

**
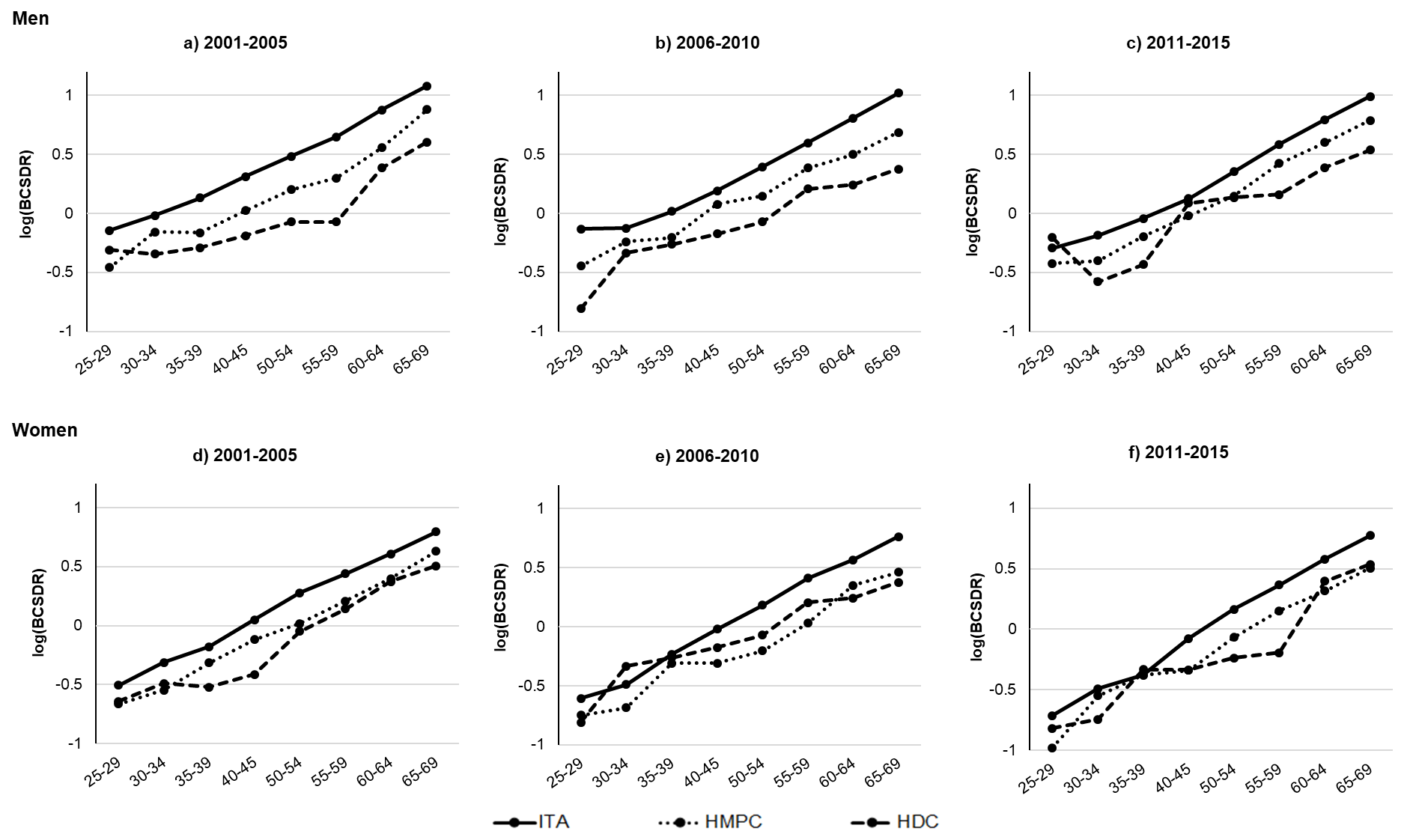
**

*Notes*: For the 2001-2005 time-period we selected the birth-cohorts from 1937 to 1976; for the 2006-2010 time-period the birth-cohorts from 1942 to 1981; for the 2011-2015 time-period the birth-cohorts from 1947 to 1986.

*Source:* Authors’ elaboration on Dynamic Rome Longitudinal Study cohort data and the Register of causes of death (ReNCaM).

**Appendix B**

**Table 1B. Gender-specific mortality HR for migrants versus Italians residing in Rome, by time-period**

|  |  | **2001-2005** | | | | | | |
| --- | --- | --- | --- | --- | --- | --- | --- | --- |
|  |  | **Men** | | |  | **Women** | | |
|  |  | **HR** |  | **95% CI** |  | **HR** |  | **95% CI** |
| **Migrant status** |  |  |  |  |  |  |  |  |
| Italian |  | 1.00 |  |  |  | 1.00 |  |  |
| Migrant |  | 0.48 | *** | (0.446-0.527) |  | 0.60 | *** | (0.546-0.653) |
| *N observations* |  | *898172* | | |  | *942659* | | |
|  |  | **2006-2010** | | | | | | |
| **Migrant status** |  |  |  |  |  |  |  |  |
| Italian |  | 1.00 |  |  |  | 1.00 |  |  |
| Migrant |  | 0.52 | *** | (0.484-0.568) |  | 0.51 | *** | (0.467-0.560) |
| *N observations* |  | *908115* | | |  | *958517* | | |
|  |  | **2011-2015** | | | | | | |
| **Migrant status** |  |  |  |  |  |  |  |  |
| Italian |  | 1.00 |  |  |  | 1.00 |  |  |
| Migrant |  | 0.64 | *** | (0.596-0.687) |  | 0.59 | *** | (0.543-0.636) |
| *N observations* |  | *898317* | | |  | *942784* | | |

*Notes:* The asterisks indicate significance *p < 0.05, **p < 0.01, ***p < 0.001. Parametric survival model with Gompertz baseline hazard and age as the time-scale.

*Source:* Authors’ elaboration on Dynamic Rome Longitudinal Study cohort data and the Register of causes of death (ReNCaM).

**Table 2B. Gender-specific mortality HR for different migrant groups versus Italians residing in Rome, by time-period**

|  |  | **2001-2005** | | | | | | |
| --- | --- | --- | --- | --- | --- | --- | --- | --- |
|  |  | **Men** | | |  | **Women** | | |
|  |  | **HR** |  | **95% CI** |  | **HR** |  | **95% CI** |
| **Origin area** |  |  |  |  |  |  |  |  |
| Italy |  | 1.00 |  |  |  | 1.00 |  |  |
| Africa |  | 0.67 | *** | (0.591-0.761) |  | 0.91 |  | (0.778-1.056) |
| Asia |  | 0.38 | *** | (0.311-0.461) |  | 0.40 | *** | (0.311-0.513) |
| Latin America |  | 0.46 | *** | (0.357-0.581) |  | 0.47 | *** | (0.364-0.595) |
| Central-Eastern Europe |  | 0.55 | *** | (0.439-0.684) |  | 0.70 | ** | (0.564-0.875) |
| HDC |  | 0.32 | *** | (0.263-0.398) |  | 0.52 | *** | (0.441-0.613) |
| *N observations* |  | *898172* | | |  | *942659* | | |
|  |  | **2006-2010** | | | | | | |
| **Origin area** |  |  |  |  |  |  |  |  |
| Italy |  | 1.00 |  |  |  | 1.00 |  |  |
| Africa |  | 0.67 | *** | (0.582-0.765) |  | 0.78 | ** | (0.648-0.931) |
| Asia |  | 0.49 | *** | (0.415-0.572) |  | 0.39 | *** | (0.313-0.494) |
| Latin America |  | 0.44 | *** | (0.340-0.573) |  | 0.43 | *** | (0.339-0.557) |
| Central-Eastern Europe |  | 0.64 | *** | (0.542-0.756) |  | 0.53 | *** | (0.441-0.630) |
| HDC |  | 0.33 | *** | (0.266-0.410) |  | 0.47 | *** | (0.387-0.562) |
| *N observations* |  | *908115* | | |  | *958517* | | |
|  |  | **2011-2015** | | | | | | |
| **Origin area** |  |  |  |  |  |  |  |  |
| Italy |  | 1.00 |  |  |  | 1.00 |  |  |
| Africa |  | 0.78 | *** | (0.687-0.894) |  | 0.96 |  | (0.812-1.139) |
| Asia |  | 0.50 | *** | (0.429-0.572) |  | 0.52 | *** | (0.439-0.624) |
| Latin America |  | 0.60 | *** | (0.481-0.740) |  | 0.56 | *** | (0.461-0.685) |
| Central-Eastern Europe |  | 0.82 | *** | (0.718-0.926) |  | 0.53 | *** | (0.460-0.604) |
| HDC |  | 0.48 | *** | (0.393-0.586) |  | 0.54 | *** | (0.449-0.659) |
| *N observations* |  | *898317* | | |  | *942784* | | |

*Notes:* The asterisks indicate significance *p < 0.05, **p < 0.01, ***p < 0.001. Parametric survival model with Gompertz baseline hazard and age as the time-scale.

*Source:* Authors’ elaboration on Dynamic Rome Longitudinal Study cohort data and the Register of causes of death (ReNCaM). Cox Model with age as the time-scale.
